# Shared velocity encoding (SVE): a new method for real-time velocity measurement with high temporal resolution

**DOI:** 10.1186/1532-429X-11-S1-O81

**Published:** 2009-01-28

**Authors:** Hung-Yu Lin, Yu Ding, YiuCho Chung, Orlando Simonetti

**Affiliations:** 1 The Ohio State University, Columbus, OH USA; 2 Siemens Healthcare, Inc, Malvern, PA USA

**Keywords:** Temporal Resolution, Echo Train Length, Respiratory Motion Artifact, Sufficient Temporal Resolution, Image Frame Rate

## Objective

To develop and demonstrate a new method for rapid, real-time, phase-contrast velocity measurement using Shared Velocity Encoding (SVE) and gradient-echo planar imaging (GRE-EPI).

## Introduction

Conventional ECG-triggered, segmented phase-contrast imaging (PC-MRI) is an accurate and clinically proven technique to characterize blood flow velocity. However, this method requires reliable cardiac gating, regular cardiac rhythm, and either signal-averaging, respiratory gating, or breath-holding to suppress respiratory motion artifacts. Furthermore, the resulting velocity information is a weighted temporal average of information acquired over multiple cardiac and respiratory cycles;short-term hemodynamic variations are lost. Real-time PC-MRI has been previously proposed using GRE-EPI [1] and spiral acquisitions [2], but limited performance has precluded routine clinical application. The aim of the present work is to design and demonstrate a novel method for rapid real-time velocity measurement with sufficient temporal resolution to eliminate the need for ECG synchronization and breath-holding, and to provide beat-to-beat hemodynamic information.

## Methods

### Sequence

SVE is a PC-MRI reconstruction technique designed to improve temporal resolution. Conventional real time PC-MRI works by alternating the polarity of velocity encoding gradients from one image frame to the next between positive (+) and negative (-) velocity encoding (i.e., [+ -], [+ -]). The velocity map is obtained by subtracting the negative velocity encoded image from the positive encoded image. The temporal resolution of the velocity map is therefore half the image frame rate. In SVE, images are acquired in the same way, but the velocity map is reconstructed by sliding the pair of images for subtraction one frame at a time (instead of two), resulting in a factor of 2 improvement in effective temporal resolution. This reconstruction technique was implemented to improve the temporal resolution of a GRE-EPI sequence for real-time PC-MRI on a 1.5 T MR scanner (MAGNETOM Avanto, Siemens, Germany).

### Imaging

Five healthy volunteers with no history of cardiovascular disease were scanned. Through-plane velocity measurements using segmented, spoiled gradient echo PC-MRI and the proposed real-time GRE-EPI with echo train length = 15 and SVE reconstruction were acquired for two slices: (i) cutting the ascending and proximal aorta and (ii) perpendicular to the distal descending aorta (2 cm superior to the renal arteries). Common acquisition parameters were: FOV = 350 × 262 mm, matrix = 160 × 120, flip angle = 25°, spatial resolution = 2.18 × 2.18 mm^2^, GRAPPA acceleration rate = 2, and Venc = 150 cm/s. The TE/TR/temporal resolution of the conventional gradient-echo and real-time GRE-EPI sequences were 3.5/7.0/42.0 ms and 2.9/14.6/58.4 ms, respectively.

### Analysis

Peak velocity measurements were compared, and Root-Mean-Square Error (RMSE) was calculated between velocity curves obtained using the real-time SVE and the conventional sequence. Linear interpolation was used prior to RMSE calculation to compare curve values at exactly the same time points.

## Results

**Figure 1:**
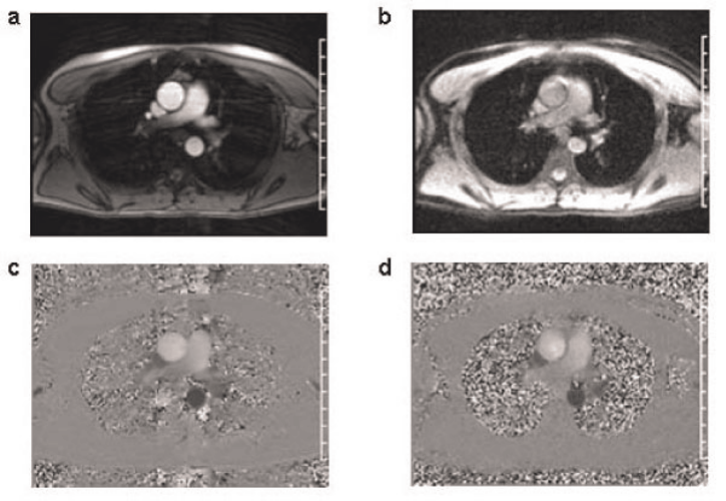
**Magnitude images acquired using (a) conventional segmented, (b) real-time GRE-EPI SVE PC-MRI, and phase velocity maps from (c) conventional segmented PC-MRI, and (d) real-time GRE-EPI SVE PC-MRI sequence in a slice cutting the ascending and descending aorta close to the aortic arch**.

*In vivo* images from one volunteer are shown in Figure 1. Magnitude images clearly show vascular anatomy in both the segmented (Figure 1a) and real-time sequences (Figure 1b). In Table 1, peak aortic velocity measurements show good agreement between conventional segmented PC-MRI and real-time PC-MRI with SVE reconstruction (*r* = 0.93, *p* < 0.05). Insignificant RMS errors (Table 1) were obtained in all regions-of-interest showing good agreement between velocity curves generated by conventional and real-time SVE techniques.

**Table 1:** Peak velocities and RMSE) of the real-time SVE technique with respect to conventional PC-MRI

ROI	Peak Velocity (Segmented)	Peak Velocity (Real-time SVE)	RMSE
Proximal Ascending Aorta	83.7 ± 4.23 cm/s	87.4 ± 5.43 cm/s	3.6153 cm/s
Proximal Descending Aorta	24.4 ± 5.61 cm/s	23.7 ± 5.39 cm/s	1.1573 cm/s
Distal Descending Aorta	20.4 ± 4.97 cm/s	20.4 ± 4.77 cm/s	1.0744 cm/s

## Conclusion

We have demonstrated the new SVE method that results in a factor of 2 improvement in effective temporal resolution in PC-MRI without sacrificing spatial resolution. With SVE reconstruction, real-time velocity measurement becomes practical with temporal resolution approaching that of conventional segmented PC-MRI.
